# Human gut metatranscriptome changes induced by a fermented milk product are associated with improved tolerance to a flatulogenic diet

**DOI:** 10.1016/j.csbj.2022.04.001

**Published:** 2022-04-05

**Authors:** Iñigo Oyarzun, Boris Le Nevé, Francisca Yañez, Zixuan Xie, Matthieu Pichaud, Gerard Serrano-Gómez, Joaquim Roca, Patrick Veiga, Fernando Azpiroz, Julien Tap, Chaysavanh Manichanh

**Affiliations:** a Microbiome Lab, Vall d’Hebron Institut de Recerca (VHIR), Barcelona, Spain; b Danone Nutricia Research, Palaiseau, France; c Molecular Biology Institute of Barcelona (IBMB), Spanish National Research Council (CSIC), Barcelona, Spain; d Centro de Investigación Biomédica en Red de Enfermedades Hepáticas y Digestivas (CIBERehd), 28029 Madrid, Spain

**Keywords:** Metatranscriptomics, Fermented milk product, Gut symptoms, Gut microbiota, Intestinal gas, *Bifidobacterium animalis* subsp. *lactis* CNCM I-2494/DN-173010

## Abstract

Healthy plant-based diets rich in fermentable residues may induce gas-related symptoms, possibly mediated by the gut microbiota. We previously showed that consumption of a fermented milk product (FMP) containing Bifidobacterium animalis subsp. lactis CNCM I-2494 and lactic acid bacteria improved gastrointestinal (GI) comfort in response to a flatulogenic dietary challenge in healthy individuals. To study the effects of the FMP on gut microbiota activity from those participants, we conducted a metatranscriptomic analysis of fecal samples (n = 262), which were collected during the ingestion of a habitual diet and two series of a 3-day high-residue challenge diet, before and following 28-days of FMP consumption.

Most of the FMP species were detected or found enriched upon consumption of the product. FMP mitigated the effect of a flatulogenic diet on gas-related symptoms in several ways. First, FMP consumption was associated with the depletion of gas-producing bacteria and increased hydrogen to methane conversion. It also led to the upregulation of activities such as replication and downregulation of functions related to motility and chemotaxis. Furthermore, upon FMP intake, metabolic activities such as carbohydrate metabolism, attributed to B. animalis and S. thermophilus, were enriched; these activities were coincidentally found to be negatively associated with several GI symptoms. Finally, a more connected microbial ecosystem or mutualistic relationship among microbes was found in responders to the FMP intervention.

Taken together, these findings suggest that consumption of the FMP improved the tolerance of a flatulogenic diet through active interactions with the resident gut microbiota.

## Graphical abstract

**Figure:**
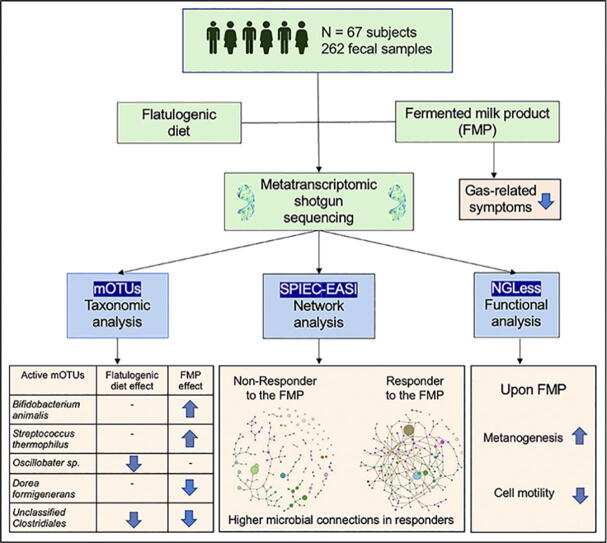

## 1 Introduction

Hydrogen, carbon dioxide, and methane are the main gases produced during the anaerobic fermentation of dietary carbohydrate residues in the large intestine [1]. Therefore, intestinal gas production will depend on both diet or amount of fermentable residues that reach the colon and colonic microbiota's composition and metabolic activity [2], [3]. Gas-related symptoms, such as abdominal bloating, distention, borborygmi, and flatulence, are experienced by up to 26% of the general population [4], [5] and up to 82% of patients with irritable bowel syndrome (IBS) [6]. Gas-related symptoms affect the quality of life and pose an economic burden for the healthcare system [6], [7].

Interest in plant-based food is increasing worldwide, because of the health benefits involved [8], [9]. However, these diets rich in fermentable residues can elicit gas-related symptoms in healthy individuals [10].

Growing evidence indicates that specific probiotic strains could help reducing bloating and/or distension in some patients with functional digestive symptoms (i.e., symptoms without identifiable cause by conventional diagnostic tests), such as the irritable bowel syndrome (IBS) [11]. Regarding individuals from the general population, a fermented milk product (FMP) containing *Bifidobacterium animalis* subsp. *lactis* CNCM I-2494 and lactic acid bacteria has been shown to improve digestive symptoms [12], [13], [14], [15]. These findings may justify the use of this FMP to enhance intestinal comfort and tolerance to plant-based diets. However, most probiotic studies have relied on metagenomics to capture the potential microbiota activity and very few on metatranscriptomic approaches, which examine the active microbial composition and metabolic functions for a better understanding of the mechanisms at play.

In the present work, we used metatranscriptomics to assess whether the observed improved tolerance of a flatulogenic diet following consumption of a FMP with *B. Lactis* CNCM I-2494 and lactic acid bacteria [16] is mediated through modulation of gut microbial activity.

We analyzed fecal samples previously taken from 67 healthy individuals enrolled in a longitudinal study exploring the effect of the FMP on microbial taxonomic profiles using a 16S rRNA gene survey [16]. Using a metatranscriptomics approach, we sought to uncover how the product affected the active gut microbial community and activated metabolic pathways, and determine which carbohydrate-active enzymes (CAZymes) are differentially expressed in response to FMP consumption. Also, we monitored the populations of bacterial species contained in the FMP before and after its consumption.

## 2 Methods

### 2.1 Study design and clinical data

The participants included in this work (n = 67), as well as their fecal samples, were part of a previous study [16]. The protocol was registered in ClinicalTrials.Gov [NCT02936713]. Participants (36 women, 31 men, 28.97 ± 7.2 years, 22.97 ± 2.4 Kg/m^2^ BMI) did not present gastrointestinal symptoms or a history of gastrointestinal disorders, and they did not take antibiotics in the two months before entering the study, thus complying with the exclusion criteria defined in the original recruitment protocol.

**Fig. 1:**
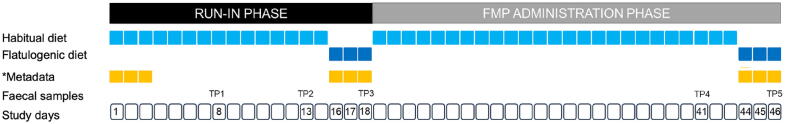
Study design describing a run-in phase of 18 days and a fermented milk product administration phase of 28 days. Habitual and flatulogenic diets are shown as light and dark blue squares, respectively. *Metadata = symptoms including the smell of gas, sensations of borborygmi, abdominal discomfort, abdominal distension, abdominal pressure or bloating, flatulence, and level of overall digestive well-being; anal gas evacuated was obtained during the day using an event counter; stool type was based on the Bristol scale. Symptoms, anal gas evacuated, and stool type were collected on days 1–3, 16–18, 44–46; stools were collected on days 8, 13, 18, 41, and 46. (For interpretation of the references to colour in this figure legend, the reader is referred to the web version of this article.)

The study was divided into two phases (Fig. 1): a run-in phase of 18 days and an FMP administration phase of 28 days. During the 28-day FMP administration phase, individuals consumed 1 pot (125 g) of the study product at breakfast and 1 pot at dinner. The study product was a fermented milk containing three *Streptococcus salivarius* subsp. *thermophilus* strains (CNCM I-2773, CNCM I-2130, CNCM I-2272), *Lactobacillus delbrueckii* subsp. *bulgaricus* (CNCM I-1519), *Bifidobacterium animalis* subsp. *lactis* (CNCM I-2494, previously referenced as DN-173010, and refered as *B. lactis* in the current manuscript), and *Lactococcus lactis* subsp. *lactis* (CNCM I-1631). The study product was manufactured and supplied by Danone Nutricia Research, Palaiseau, France and contained per g at least 3.4 × 10^7^ colony forming units (cfu) of *B. lactis*, 1 × 10^6^ cfu of *L. lactis*, and 1 × 10^7^ cfu of *S. thermophilus* and *L. bulgaricus*. During each phase, the individuals ate their habitual diet, except for the last three days when a flatulogenic diet (61% carbohydrates, 25% proteins and 14% fat, 27 g of fiber per day) was administered.

Fecal samples were collected at five time points: days 8, 13, 18 (during run-in phase) and days 41, 46 (during administration phase). Gas-related symptoms were recorded at different time points (see Fig. 1 under the metadata label) through a questionnaire filled in by each participant. These symptoms included smell of gas, sensations of borborygmi, abdominal discomfort, abdominal distension, abdominal pressure or bloating, flatulence and level of overall digestive well-being. All symptoms were ranked on a scale from 0 to 10, except fo digestive well-being, which was ranked from −5 to + 5. A classification of the stool consistency, based on the Bristol stool scale, was also included in the questionnaire. Individuals were asked to count the number of anal gas evacuations per day using an event marker (Hand Tally Counter No 101, Digi Sport Instruments, Shangqiu, China).

### 2.2 From stool to RNA shotgun sequences (metatranscriptomic)

Stool samples were collected at five time points but not all participants brought samples at each time point (Supplementary Table S1). The missing samples were mostly due to omission or personal reasons of the participants. A total of 262 fecal samples were processed through total RNA extraction, DNase treatment, quality control of the treated RNA and rRNA removal, and cDNA synthesis for shotgun sequencing following the Illumina TrueSeqBiot protocol. A total of 1.79E10 pair-ended sequences of 150 nucleotides was generated. Seven samples that did not pass the quality cut-off after RNA extraction were excluded from further analysis.

### 2.3 Sequence preprocessing

The KneadData software (https://huttenhower.sph.harvard.edu/kneaddata) was used to decontaminate the raw metatranscriptomic sequence data. This tool removed low quality reads using Trimmomatic [17] with a sliding window of 4:20, sequence adapters, human RNA and prokaryotic ribosomal RNA. The number of sequences remaining was 1.152E + 10 (64%).

### 2.4 Sequence processing

The mOTU software (version 2.5), which is based on housekeeping marker genes, was used to recover the taxonomic profiles at the species level on absolute abundance or raw counts [18]. A programming language called NGLess was used to build the functional profiles [19]. To functionally annotate the reads, they were mapped against the Integrated Gene Catalog (IGC) [20], which is a non redundant human gut microbiome database integrated into NGLess. During the mapping step, the minimum match size of the query sequence was set up at 100 nucleotides and 95% identity. These parameters were chosen after optimizing for sensitivity and specificity with a subset of samples. To this end, different combinations of minimum match size and identity were tested and the mapping rates were taken into account. NGLess processing code can be found in Supplementary Method 1. The functional profiles were retrieved in raw counts for the IGC gene IDs and KEGG orthology (KO) IDs. Gene orthologs from *Bifidobacterium animalis* subspecies lactis CNCM I-2494 were retrieved by selecting the best hit from a BLAST analysis.

### 2.5 Statistical analysis

Statistics on taxonomic profiles were performed using ANCOM-II [21], a methodology that considers the excess of zeroes in microbiome data, identifying and classifying them into three sources: namely outlier zeroes, structural zeroes and sampling zeroes. Marker gene-based operational taxonomic units (mOTUs) with<10% prevalence across samples were excluded from the analysis.

The functional analysis was performed using KO annotations, Metagenomic Species Pan-genomes (MSP) analysis and carbohydrate-active enzyme (CAZy) annotations. Using the KO annotation profiles obtained from NGLess, we performed a differential expression (DE) analysis comparing different sampling time points to address various questions of interest. These questions, using both taxonomic and functional profiles, were related to the flatulogenic diet and the effects of the FMP and were addressed by comparing TP2 vs. TP3 and TP3 vs. TP5, respectively (Fig. 1). The DE analysis was computed using the Bioconductor package limma [22] with the limma-voom approach. We adjusted the unwanted variation of the model for the known covariate SubjectID, taking into account the longitudinal feature of the study, and for unknown sources of variation using surrogate variables with the R package SVA [23]. The t-statistics of each KO resulting from these models were then used to perform a fast gene set enrichment analysis (FGSEA) [24], which provided the enriched KEGG pathways between two time points. NGLess output files were normalized within samples adjusted by the gene length.

For the between-sample normalization, we used the Trimmed Mean of M-values (TMM) [25], one of the highest performing normalization methods [26]. Also, once the normalization factor for the library sizes was calculated, the data were normalized to counts per million (CPM) and were transformed into logarithmic data in base 2. KOs were filtered out by their expression using the filterByExpr() function from the edgeR package [27], [28].

To evaluate the gene expression of the different species present in the FMP, their genes were isolated based on the work developed by Plaza-Oñate et. al., where MSPs were reconstituted aligning 3143 genomes against the IGC catalog and using MSPminer [29]. Only *Bifidobacterium animalis* and *Streptococcus thermophilus* were evaluated, as the other FMP species were detected in a very low number of samples. Samples in which the species were not present based on the taxonomic profile were discarded and the IGC gene counts of each sample were normalized by the abundance of the species. Metatranscriptomic data were normalized by mOTU abundance, which allowed the detection of genes truly differentially expressed and not due to taxonomic variation. For between-sample analysis, samples were also normalized by TMM but this time the IGC genes were filtered by a minimum mean abundance of 0.01% and a minimum prevalence of 10% across samples. DE analysis was then performed using limma-voom.

MSP analysis was also performed for *M. smithii* using IGC genes belonging to each of the M. smithii’s MSPs (msp_0560 and msp_0871). After isolating the IGC genes belonging to the MSP, samples with no presence of M. smithii mOTU were discarded and the remaining (131 samples) were normalized by the mOTU abundance in each sample. Then, IGC genes were converted to KO annotations through a functional map. The functional map was created using the eggnog 5.0 mapper software on the IGC database [30], [31]. The normalization and DE analysis were performed as above mentioned. The t-statistics resulting from the DE analysis were introduced into a GSEA to see which KEGG pathways are enriched.

To study the activity of CAZymes using the CAZy database, the above functional map was also used to identify the IGC genes with CAZyme class annotations, and a DE analysis was performed. For this analysis, the TMM normalization method and the filtering by abundance and prevalence approach were also applied.

### 2.6 Association between symptom records and taxonomic and metabolic activities profiles

Metadata variables were associated with mOTU abundance using the MaAsLin2 R package [32]. MaAsLin2 was run with the default parameters, except for the minimum abundance parameter, which was set at 0.0001. TMM was chosen as the normalization method. To account for multiple comparisons of all taxa, false discovery rate (FDR) correction was used, and the results were considered significant when the adjusted P value was below 0.05. Also, Subject ID was used as a random effect to account for the longitudinal feature of the study. Due to the study design, no symptoms associated with the TP4 sampling point were recorded (i.e., after habitual diet + FMP consumption). Samples collected at TP4 were therefore discarded from these analyses.

### 2.7 Network analysis

Inference of microbial ecological networks was performed using the SPIEC-EASI (Sparse Inverse Covariance Estimation for Ecological Association Inference) software [33]. This approach builds microbiome networks using sparse neighborhood and inverse covariance selection algorithms. The networks were constructed with a minimum sparsity/lambda parameter of 1e-2 and using Meinshausen and Bühlmann's neighborhood selection method [34]. We isolated the samples for TP2 (n = 56), TP3 (n = 48) and TP5 (n = 52) separately. mOTUs with a prevalence of at least 20% across the subset of samples and a minimum abundance of 0.1 % were used as SPIEC-EASI input. The edges in red indicate a negative relation and green a positive relation. Gas-producing bacteria were marked with a black halo and the size of the nodes is proportional to the mean relative abundance of each mOTU.

### 2.8 Classification of response to FMP

For a participant to be classified as a responder, the frequency of his/her anal gas evacuation should be lower at TP5 vs. TP3. To this end, participants with samples collected at TP2, TP3, and TP5 were isolated (n = 39 individuals, 28 responders and 11 non-responders, totaling 117 samples).

Networks were constructed using the same parameters as above. However, in this case there are much more samples available for the responder group and this may bias the generation of bacterial connections. Therefore, we performed network analysis using an equal number of responders and non-responders using 100 iterations for each group. To do so, we randomly selected seven individuals from each group and then pooled the samples of the three time points (TP2, TP3, and TP5) of each individual for each group, since no significant difference between time points was observed. In this way, we obtained 21 samples for both responder and non-responder groups. We then re-computed the degrees for each iteration.

## 3 Results

### 3.1 Effect of the flatulogenic diet and FMP on gas-related symptoms and anal gas evacuation

As described in our previous work, data related to gas-related symptoms, anal gas evacuated, and stool types were collected at the beginning and at the end of a run-in phase (days 1–3 and 16–18) and at the end of a FMP administration phase (days 44–46) (Fig. 1). Previous analysis of these data showed that a high-residue diet (flatulogenic diet) induced gas-related symptoms, increased the daily evacuation of anal gas, and impaired digestive well-being as compared to a habitual diet, and that FMP intake improved the tolerance of the flatulogenic diet [16]. To correlate these clinical data with microbiome data, stools were also collected at baseline under habitual diet (TP1 and TP2), after the flatulogenic diet challenge (TP3), during FMP intake (TP4), and after a second flatulogenic diet challenge (TP5).

### 3.2 Effect of the flatulogenic diet and FMP consumption on microbial alpha- and beta-diversity

No significant differences were found in microbial alpha-diversity between all the time points in a pairwise manner, as assessed by the Chao1 (richness) and Shannon (richness and evenness) indexes, except between TP2 and TP3 using the Chao1 index (n = 46, Wilcoxon signed-rank test, p = 0.026, Supplementary Fig. S1) where the flatulogenic diet was associated with a reduction in gut microbial richness. This result was not reproduced between TP4 and TP5, suggesting that consumption of the FMP prevented this flatulogenic diet-induced reduction in microbial richness.

Beta-diversity (between samples) analysis, based on unweighted UniFrac metrics computed on taxonomic profiles, showed higher compositional variability between TP1 and TP2 (two samples taken during the run-in phase upon habitual diet) than between TP3 (taken during the first flatulogenic challenge) and TP4 (taken during the FMP administration phase) (Supplementary Fig. S2). Interestingly, the overall time point comparisons using the Friedman test showed a trend towards variability of gut microbiome composition related to the distinct dietary interventions (p = 0.07 for unweighted UniFrac and p = 0.19 for weighted UniFrac distances), thereby pointing to an overall dynamic microbiome community throughout the study.

### 3.3 Effect of the flatulogenic diet and FMP consumption on active microbiome composition

**Fig. 2:**
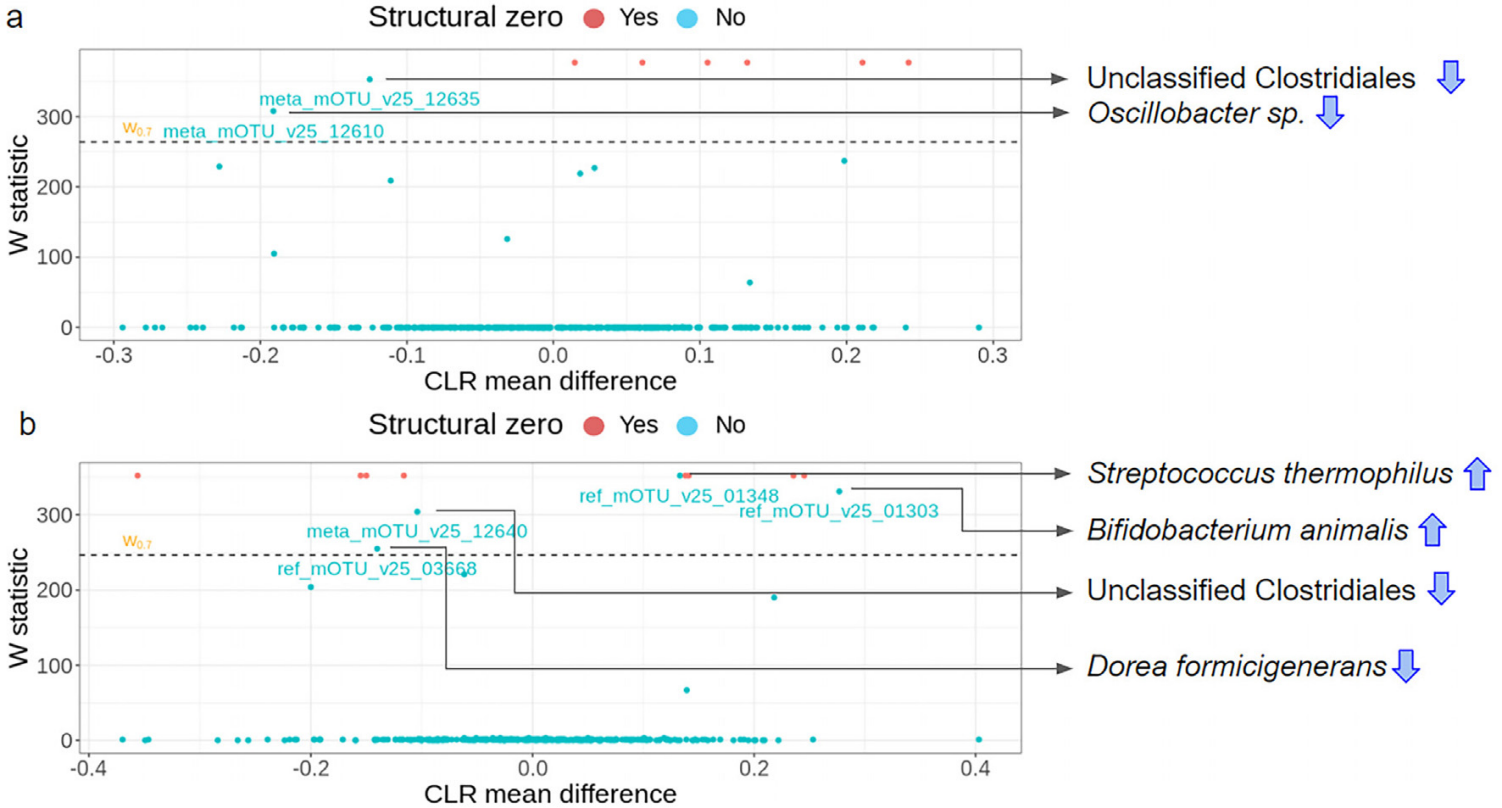
Comparison of taxonomic profiles shown by volcano plots produced by the ANCOM-II software. a) Comparison between the habitual diet (TP2) and flatulogenic diet (TP3). The blue arrows indicate a depletion of mOTUs after the flatulogenic diet. b) Comparison between the flatulogenic diet (TP3) and flatulogenic diet following 28 days of FMP intake (TP5). The blue arrows indicate an enrichment or depletion of mOTUs after the flatulogenic diet + FMP. Blue dots over the dashed line indicate species (mOTUs) with significant and differential abundance, while the red dots are mOTUs classified as structural zeroes (could be considered false positives). (For interpretation of the references to colour in this figure legend, the reader is referred to the web version of this article.)

A differential abundance (DA) analysis of the microbiome data using ANCOM II indicated that the flatulogenic diet was associated with a significant depletion of two bacterial species from the Clostridiales order: Oscillibacter sp. (meta_mOTU_v25_12610) and an unclassified species (meta_mOTU_v25_12635) (Fig. 2a).

FMP intake was associated with significant enrichment of *Streptococcus thermophilus* (ref_mOTU_v25_01348) and *Bifidobacterium animalis* (ref_mOTU_v25_01303), two of the bacterial species present in the FMP, and with a significant decrease in *Dorea formicigenerans* (ref_mOTU_v25_03668) and an unclassified species from the Clostridiales order (meta_mOTU_v25_12640) (Fig. 2b). *Lactobacillus delbrueckii* (ref_mOTU_v25_01298), another bacterial species in the FMP, was detected after consumption of the FMP (TP4 and TP5). Interestingly, a metatranscriptomics approach indicated that most FMP species were detected or found enriched upon product consumption, suggesting the species' survival and potential metabolic activity in the GI tract (Supplementary Fig. S3).

**Fig. 3:**
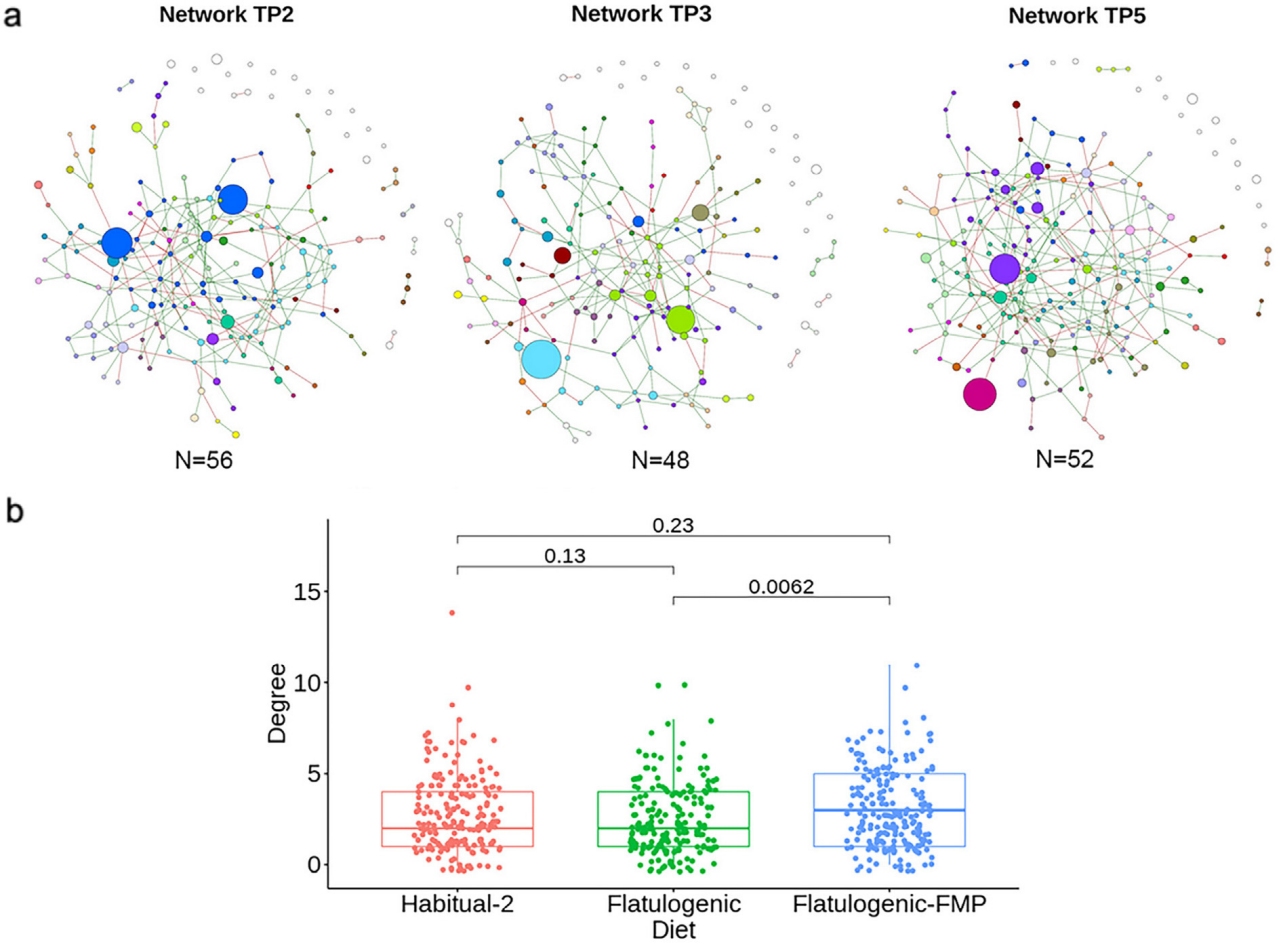
Microbiome network analysis using the mOTU table. a) Each network represents samples collected at each of the following time points (TP2, TP3, and TP5). A higher resolution of the labeled networks is available in Supplementary Fig. S7. Nodes represent microbial species, and edges are links between each node; red edges indicate negative covariation and green edges positive covariation between species. The size of the nodes is proportional to the mean relative abundance of each mOTU and the colour to the network clusters. b). Plotting of degrees, which are the number of links between each species, for the three time points. TP2 = baseline; TP3 = flatulogenic diet; TP5 = flatulogenic diet + FMP. (For interpretation of the references to colour in this figure legend, the reader is referred to the web version of this article.)

We used a network-based analytical approach to understand the complex interactions between microbiota members, applying the SPIEC-EASI statistical method, which provided microbiome networks (Fig. 3a). Analysis of the network degree distributions (Fig. 3b) for each time point showed that FMP intake led to a significant increase in microbial interactions (Mann-Whitney test, p = 0.006). Interestingly, *B. animalis,* which did not present any interactions with other bacteria at baseline (TP2), was connected to three other species (Clostridiales species incertae sedis, *Flavonifractor plautii* and Ruminococcaceae gen. incertae sedis) after the flatulogenic diet challenge (TP3), and to an unclassified Eggerthellaceae species after FMP intake (TP5). S*. thermophilus* was detected and connected to *Streptococcus* sp. only at TP5. It was not included in the network at the other time points, which could be explained by a much lower abundance and prevalence.

### 3.4 Effect of the flatulogenic diet and FMP consumption on gut metatranscriptome

To specifically study the expression of genes from the two main FMP species (*B. lactis* and *S. thermophilus*), we isolated their genes assigned to *B. animalis* and *S. thermophilus* from the Metagenomic Species Pan-genomes (MSP) [29]. Read counts, normalized by the species abundance, for each gene transcript and sample were plotted on a heatmap (Supplementary Fig. S4). Gene transcripts assigned to both species increased upon FMP consumption (i.e., at TP3 vs TP5). We detected 38 and 90 genes assigned to *B. animalis* and *S. thermophilus*, respectively, for which their expression was increased by > 1.5 times at TP5 compared to TP3 (FDR < 0.05). Among the B. animalis genes, 68% (29) belong to the 10% most expressed genes of *B. lactis* in mice [35] (Supplementary Table S2). These findings confirm that *B. lactis* and *S. thermophilus* survived to the GI tract, are transcriptionally active in the gut and possibly to adapt to their environement.

**Fig. 4:**
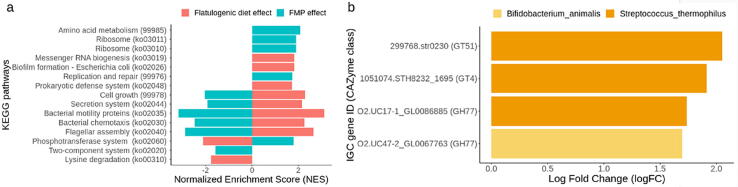
Effects of the flatulogenic diet and FMP intake on microbial activity. a) The effect of the flatulogenic diet alone on KEGG pathways was studied by comparing samples collected at TP2 vs. TP3. The effect of the flatulogenic diet combined with the FMP administration phase was evaluated by comparing samples collected at TP3 vs. TP5, b) FMP associated CAZyme activities during flatulogenic diet. GH: glycoside hydrolases; GT: glycosyltransferases.

We further explored the variations of the metatranscriptiome for the resident community. The flatulogenic diet challenge was also associated with increased microbial activity of KEGG pathways involved in virulence and colonization: cell motility, such as flagellar assembly (ko02040), bacterial motility proteins (ko02035) and bacterial chemotaxis (ko02030), as well as pathways related to biofilm formation (ko02026), the prokaryotic defense system (ko02048), the secretion system (ko02044), and cell growth (#99978) (FDR < 0.1). Meanwhile, pathways leading to lysine degradation (ko00310) and phosphotransferase system (ko02060) were downregulated (Fig. 4a) (FDR < 0.1).

Remarkably, these analyses also revealed that FMP intake was associated with reverse effects on the pathways related to motility and chemotaxis. Moreover, upon FMP intake, pathways related to amino acid metabolism (#99985), ribosome (ko03011), and replication and repair (#99976) were upregulated (Fig. 4a). These results indicate that a flatulogenic diet might increase cell motility and cell growth and reduce amino acid degradation, whereas FMP consumption exhibited the opposite effect.

Moreover, analyzing the carbohydrate enzymatic activities (CAZyme classes) among the whole ecosystem, FMP intake was associated with significant changes in, glycoside hydrolases (GHs) and glycosyltransferases (GTs) (Fig. 4b). Noticeably, these activities belonged to *S. thermophilus* or *B. animalis* MSPs. Three CAZyme families (GT4, GT51, and GH77) belonging to *S. thermophilus* and one (GH77) belonging to *B. animalis* were found to be upregulated. GHs hydrolyze the glycosidic bond between two or more carbohydrates, and GTs form glycosidic bonds by transferring sugar moieties from activated donor molecules to other sugar molecules. These results suggest that FMP bacterial species, under a flatulogenic diet condition, activated specific enzymes that could be responsible for the breakdown of carbohydrate-based structures.

### 3.5 Association of symptoms with microbiome activity profiles

As our previous study revealed that FMP intake was associated with a reduction of gas-related symptoms caused by the flatulogenic diet [16], we examined the association between symptoms and microbial taxonomic and functional profiles. We did not observe a significant association between taxa and symptoms using a generalized linear model-based tool (MaAsLin2) on taxonomic tables. However, applying MaAsLin2 on functional profiles, four CAZyme activities showed a negative association with symptoms. Several CAZyme families including CBM48, GH88 and GH77 were negatively associated with the sensation of borborygmi and the latter pathway was also negatively associated with a sensation of abdominal pressure or bloating (FDR < 0.05) (Supplementary Fig. S5). These findings suggest that FMP consumption improves the tolerance of a flatulogenic diet by enhancing carbohydrate metabolism.

### 3.6 Responders and non-responders to the FMP

**Fig. 5:**
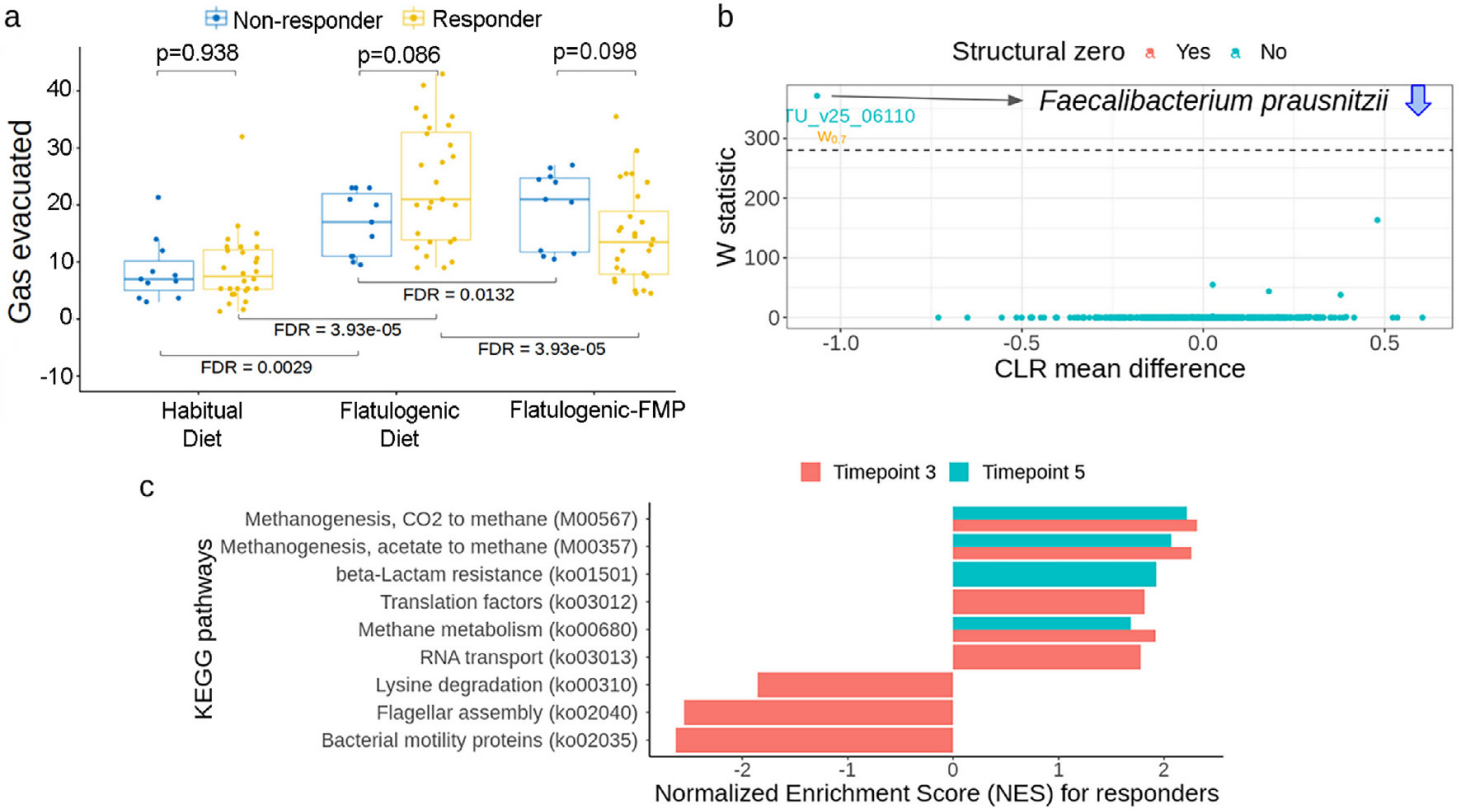
Responder vs. non-responder groups to FMP based on the frequency of anal gas evacuated. a) Classification into responder and non-responder groups to FMP at baseline (TP2), before (TP3) and after FMP intake (TP5). b) Comparison between responder and non-responder groups at the taxonomic level using ANCOM. Only significant results obtained at TP5 are plotted. c) Comparison between responder and non-responder groups at the functional level (KOs) using the limma-voom approach and performing a fast gene set enrichment analysis (FGSEA) at TP3 and TP5.

We hypothesized that not every participant would experience an improvement of gas-related symptoms upon FMP intake. Therefore, we categorized as responders in whom consumption of the FMP produced a reduction in the number of daily anal gas evacuations (i.e., with a lower number of gas evacuated at TP5 vs. TP3; see Methods section). As expected, the classification of individuals on the basis of responder status revealed a significant decrease in the frequency of anal gas evacuations in the responder group (n = 28) and an increase in the non-responder group (n = 11) after FMP intake (Fig. 5a). Using this classification, we observed that following FMP consumption, responders reported a significant decrease in other gas-related symptoms, such as the smell of gas, sensations of borborygmi, abdominal distension, abdominal pressure or bloating and flatulence, and an increased level of overall digestive well-being. In contrast, non-responders did not present significant changes for the other gas-related symptoms (Supplementary Fig. S6). Due to the approach we used to define responder and non-responder, we cannot, for an obvious reason, interpret the differences between responder and non-responder in terms of anal gas evacuated.

A taxonomic profile comparison showed that responders had a lower relative abundance of *Faecalibacterium prausnitzii* (mOTU_v25_06110) vs. non-responders after FMP intake (Fig. 5b). After the flatulogenic diet (TP3), functional profile comparison showed that bacterial motility and lysine degradation were downregulated in the responder group, whereas several activities, including methanogenesis, were upregulated compared to the non-responder group (limma-voom, FGSEA analysis, q < 0.001, Fig. 5c). After FMP intake (TP5), the responder group showed higher methanogenesis activity than non-responders (q < 0.05, Fig. 5c).

Since methanogenesis was found upregulated on responders and *Methanobrevibacter smithii* is known as the main producer of methane [36], we hypothesized that this species has an increased metabolic activity. Using MSP minner, we uncovered two MSPs identified as *M. smithii* (msp_0560 and msp_0871) and performed a DE and GSEA analyses. We observed that oxidative phosphorylation and methanogenesis pathways were among the top enriched pathways of msp_0560 (FDR < 0.05, Supplementary Table S3).

**Fig. 6:**
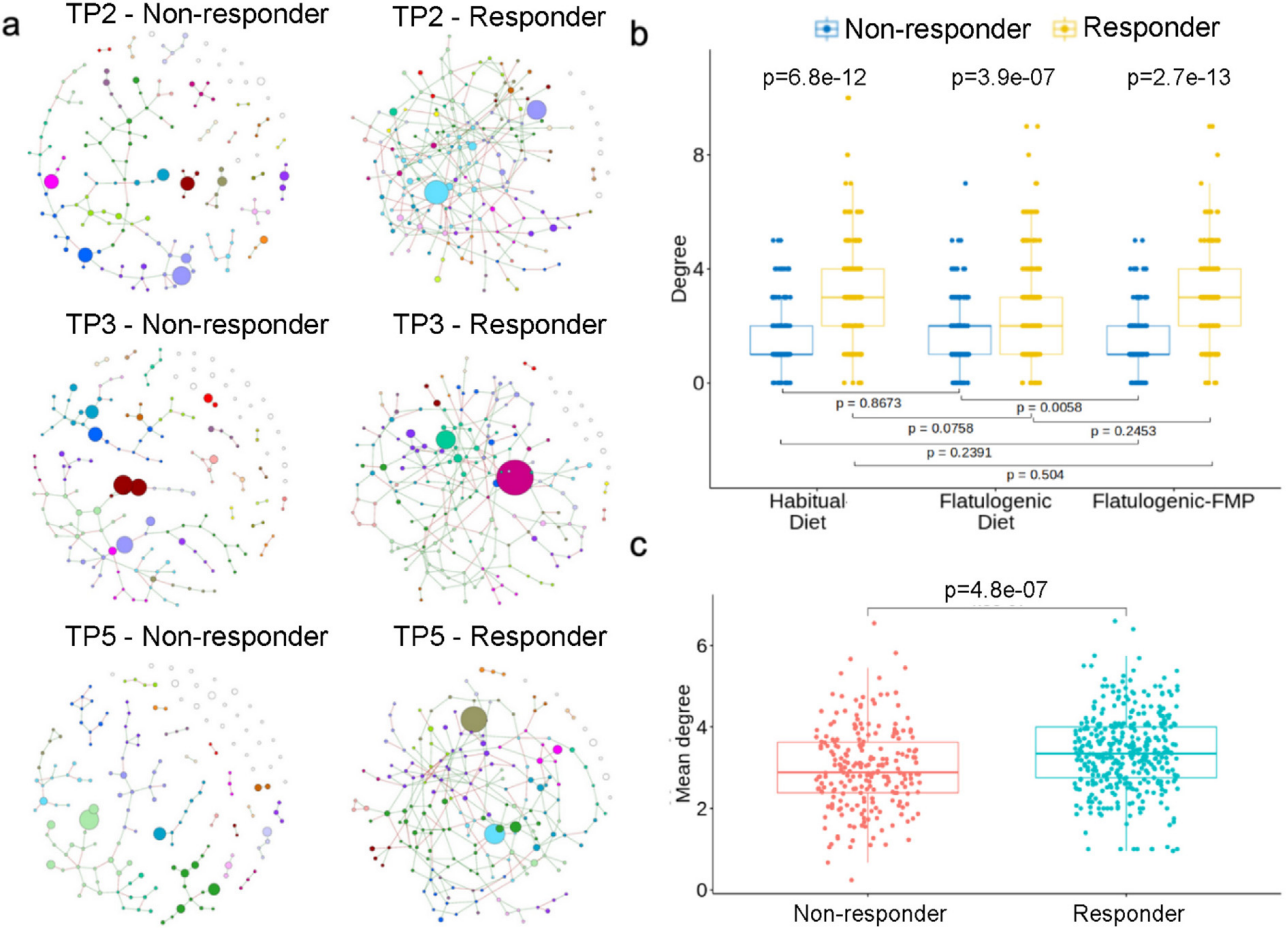
Microbiome network analysis of responders (n = 28) and non-responders (n = 11) to the FMP. a) Network representation for responder and non-responder groups at three time points. A higher resolution of the labeled networks is available in Supplementary Fig. S8. b) Distribution of degrees (connections) between responders and non-responders over the three time points. c) Mean degrees obtained from randomization of an equal number of samples from each group to avoid bias that could be introduced by a different number of samples in each group and therefore a different number of processed mOTUs.

Network analysis based on the mOTU table and comparison of responders (n = 28) vs. non-responders (n = 11) through the three time points (TP2, TP3 and TP5) revealed that responders showed higher connections between bacteria than non-responders at the three time points (p = 6.8E-12, p = 3.9E-07, p = 2.7E-13, respectively, Fig. 6a, b). However, no differences were found comparing between time points within responders. Taken together, these findings suggest the number of connections between bacteria was dependent on responder status but not on the different interventions (flatulogenic diet and FMP intake). Nevertheless, as the number of participants could affect the number of bacterial connections, the degree distribution for 21 samples (7 individuals) of each group was computed over 100 iterations (see Methods section). Responders continued to display significantly higher connections than non-responders (Fig. 6c). Taken together, these findings indicate that participants who responded positively to the FMP, showing a reduction in gas-related symptoms, harbored a more connected microbial ecosystem compared to non-responders.

## 4 Discussion

In the present work, we used metatranscriptomics to assess whether the improved tolerance of a flatulogenic diet following consumption of a FMP with *B. lactis* CNCM I-2494 and lactic acid bacteria is mediated through modulation of gut microbial activity. Previous research using metatranscriptomics on human fecal samples to investigate the effect of probiotic strains on the gut microbiome has been done with few individuals [35], [37]. To the best of our knowledge, the present study is the first to apply metatranscriptomics to a larger cohort and in a longitudinal framework to evaluate the effect of a FMP on the taxonomic profile and metabolic activity of the human gut microbiome.

With respect to the species present in the FMP, our results show that the relative abundance of *B. animalis*, *S. thermophilus, L. delbrueckii* and *L. lactis* increased upon FMP intake. The observation that several of the FMP species were resistant to gastric acid, were able to survive along the GI tract down to the colon and were involved in carbohydrate metabolism at least during the FMP intake period is in line with previous reports [35], [38].

Interestingly, *L. delbrueckii* and *L. lactis* did not appear when comparing TP3 and TP5 using the ANCOM method (Fig. 2b). Their absence may be explained by a lower prevalence and abundance than the two other species, therefore not captured by the ANCOM tool (Supplementary Fig. S3).

The flatulogenic diet led to a significant increase in gas-related symptoms in all participants [16]. At the active microbiota level, it led to a reduction in microbial richness, a significant decrease in two Clostriadiales taxa, and an overexpression of metabolic pathways related to cell motility and growth. The observation that the flatulogenic diet was associated with a loss of microbial richness disagrees with one of our previous studies [39]. The discrepancy between the studies could be related to the higher number of individuals included in the present work (n = 46 vs. n = 20) or to the different metaomics approaches (DNA vs. RNA). Also, the fact that a second flatulogenic diet challenge following FMP consumption did not affect microbial richness suggests that the FMP can prevent a loss of diversity. Interestingly, the reaction of responders to the flatulogenic diet appeared to be stronger than that of non-responders in terms of borborygmi, abdominal distention and sensation of flatulence. Although the difference was not significant, a trend could be appreciated, suggesting that responders are more sensitive to the diet challenge and have a more dynamic gut physiology (Supplementary Fig. S6). At the functional level, the flatulogenic diet, which consisted of a high percentage of carbohydrates (61%) and a low percentage of fat (14%), was associated with metabolic pathways related to cell motility, chemotaxis, and cell growth. Of note, in these analyses, cell growth might be related to cell size rather than cell replication, as the KEGG database was originally developed for Eukaryotes. In a habitual diet, as we previously evaluated in a cohort of 84 healthy Spanish volunteers using a 24-h diet recall [40], individuals showed an average consumption of 55% carbohydrate and 20% fat. Altogether, our findings suggest that bacterial populations adapt to a higher carbohydrate and lower fat intake by enhancing motility and growth activities. A previous study on *E. coli* reported that motility is upregulated during growth in poor carbon sources, while it is downregulated in rich carbon sources to enable higher investment in biosynthetic machinery for replication [41]. Our findings report the behavior of members of an ecosystem that may require a different adaptation strategy to cope with stress or an extreme nutritional condition. This disagreement calls for further investigation using a more complex microbial community than *E. coli* alone.

After FMP intake, aside from a reduction of all gas-related symptoms [16] and a differentially higher relative abundance of *B. animalis* and *S. thermophilus*, we observed a significant increase in carbohydrate metabolism attributed to the two FMP species, a decrease in *Dorea formicigenerans* and an increase in methanogenesis with *M. smithii* as the main contributor among responders to the FMP. First, these results suggest that FMP bacterial species activated specific enzymes responsible for the breakdown of carbohydrate-based structures from the flatulogenic diet. The capacity of FMP species to perform this function has been previously demonstrated [35]. Moreover, as *D. formicigenerans* is a major hydrogen producer [42], we presume that the action of the FMP was associated with changes in the gut ecosystem towards a conversion of hydrogen to methane, thereby reducing the volume of intestinal gas from 4 to 1 fold [43], which is in agreement with a previous study showing a reduction of breath hydrogen after FMP consumption [16]. This hypothesis agrees with our finding of a decrease in the frequency of anal gas evacuations upon FMP intake.

Moreover, upon FMP intake, the individuals showed an upregulation of carbohydrate metabolism, which was also negatively associated with gas-related symptoms such as the sensation of borborygmi and abdominal bloating. This carbohydrate metabolism activity may contribute to metabolizing the excess of carbohydrate ingested. Finally, our findgings suggest that FMP intake was associated with an opposite functional effect compared to the flatulogenic diet, as members of the ecosystem were able to focus more on a replication and repair strategy activated during the FMP intake than on a nutritional stress imposed by an extreme diet condition [44].

It should be noted that our study has several limitations. Although mOTUs2 software claims a high correlation between metatranscriptomic and metagenomic data, the normalization by species abundance step would have been more accurate having both types of data to test how the expression profile of species changed over the study. Other limitations of this exploratory study is the lack of a control group during the FMP administration phase and an absence of assessment of the potential influence of an habitual diet on the response to the FMP.

Furthermore, the analyses were done at the species level and not at the strain level, as we opted for mOTUs2 software, which handles analysis down to the species level. Then, it is important to remark that this work is a post-hoc analysis of a previous clinical study that was not specifically designed for metatranscriptomics analysis. All the results reported herein are to be considered only as hypothesis-generating. Finally, the higher variability between TP1 and TP2 (both on similar diet conditions) compared to TP3 and TP4 (on different diet conditions) could be explained by a possible change in participants' behavior, particularly in the choice of their habitual diet, caused by their participation in a longitudinal study. However, this cannot be confirmed as dietary intake outside of flatulogenic diet periods was not monitored.

With the primary assumption that not every individual responds in the same way to the FMP, we discovered that the responder group behaved differently compared to non-responders, with a higher level of gene expression related to methanogenesis. Also, the network theory emerges as a promising approach for modeling complex biological systems with multifaceted interactions between members of a community, such as the microbiota. Using the network analysis approach, we uncovered that responders presented a more connected microbial ecosystem upon FMP intake, reflecting a more dynamic and adaptable microbial community and predisposing them to better usage of the FMP species to reduce gas-related symptoms.

Taken together, these results suggest that FMP consumption of a FMP with *B. lactis* CNCM I-2494 and lactic acid bacteria help mitigate gas-related symptoms elicited by a flatulogenic diet in healthy volunteers by indirectly reducing the volume of gas and generating more complex interactions between commensals of the gut microbiota.

## 5 Declarations

### 5.1 Ethics approval and consent to participate

Participants were recruited by public advertisement and received a monetary compensation. The fermented milk product were provided by the investigators. The protocol was approved by the Institutional Review Board of University Hospital Vall d’Hebron and all participants gave written informed consent prior to inclusion.

### 5.2 Consent for publication

All authors read and approved this version of the manuscript for publication.

### 5.3 Availability of data and material

Sequence data and metadata were uploaded in the Embl nucleotide archive with the following accession number: PRJEB51894.

## Funding

This research was supported by a grant from Danone Nutricia Research. Danone Nutricia Research authors participated in the study design, interpretation of the data and in the writing of the report. Francisca Yáñez was supported by a fellowship from ANID, BECAS Chile, No. 72190278. Zixuan Xie received a fellowship from the European Union’s Horizon 2020 research and innovation program under the Marie Sklodowska-Curie Action, Innovative Training Network: FunHoMic; grant number 812969. CIBERHED is funded by the Instituto de Salud Carlos III.

## Authors contributions

BLN, FA, CM designed the study and obtained the resources to carry out the assessment. FY processed the samples for sequencing. IO, GSG, ZX, JT, CM processed, analyzed and interpreted the data. IO and CM wrote the manuscript. BLN, FA, IO, GSG, ZX, JT, PV, FY, JR, CM authors helped in manuscript preparation and read and discussed the results and conclusions included. All authors read and approved the final version of the manuscript.

## Declaration of Competing Interest

The authors declare the following financial interests/personal relationships which may be considered as potential competing interests: BLN, JT, VP, and MP are Danone Nutricia Research employees. FA and CM have received research grants from Danone Nutricia Research. Other authors have nothing to disclose.
