# Supplementary material for: Human gut metatranscriptome changes induced by a fermented milk product are associated with improved tolerance to a flatulogenic diet
Source: Comput Struct Biotechnol J. 2022 Apr 5;20:1632–41. doi: 10.1016/j.csbj.2022.04.001 (PMC9014321; doi:10.1016/j.csbj.2022.04.001)
Supplement: Supplementary data 1 [file mmc1.pdf]

```
#!/usr/bin/env ngless
ngless "1.0"
import "mocat" version "1.0"
import "igc" version "1.0"
local import "motus" version "2.5"

input = load_mocat_sample(ARGV[1])
RESULTS = ARGV[2]

igc_mapped = map(input, reference='igc', mode_all=True)



########################
########################

igc_mapped_post = select(igc_mapped) using |mr|:
    mr = mr.filter(min_match_size=100, min_identity_pc=95, action={drop})
    if not mr.flag({mapped}):
        discard

mapstats_post = mapstats(igc_mapped_post)
write(mapstats_post,
      ofile=RESULTS </> 'post_mapstats_min100_ide95.txt',
      auto_comments=[{hash}, {script}])

igc_counts = count(igc_mapped_post,
                    features=['KEGG_KOs'],
                    multiple={dist1},
                    normalization={scaled})
write(igc_counts,
       ofile=RESULTS </> 'KEGG_min100_ide95.counts.txt',
       auto_comments=[{hash}, {script}])

igc_counts = count(igc_mapped_post,
                   features=['seqname'],
                   multiple={dist1},
                   normalization={scaled})
write(igc_counts,
      ofile=RESULTS </> 'IGC_seqname.counts.txt',
      auto_comments=[{hash}, {script}])

########################
########################
```
